# Langevin based turbulence model and its relationship with Kappa distributions

**DOI:** 10.1038/s41598-022-05996-0

**Published:** 2022-02-08

**Authors:** Iván Gallo-Méndez, Pablo S. Moya

**Affiliations:** Departamento de Física, Facultad de Ciencias, Universidad de Chile, Santiago, Chile

**Keywords:** Fluid dynamics, Statistical physics, thermodynamics and nonlinear dynamics

## Abstract

Kappa distributions (or $\kappa$-like distributions) represent a robust framework to characterize and understand complex phenomena with high degrees of freedom, as turbulent systems, using non-extensive statistical mechanics. Here we consider a coupled map lattice Langevin based model to analyze the relation of a turbulent flow, with its spatial scale dynamic, and $\kappa$-like distributions. We generate the steady-state velocity distribution of the fluid at each scale, and show that the generated distributions are well fitted by $\kappa$-like distributions. We observe a robust relation between the $\kappa$ parameter, the scale, and the Reynolds number of the system, Re. In particular, our results show that there is a closed scaling relation between the level of turbulence and the $\kappa$ parameter; namely $\kappa \sim \text {Re}\,k^{-5/3}$. We expect these results to be useful to characterize turbulence in different contexts, and our numerical predictions to be tested by observations and experimental setups.

## Introduction

Turbulence is an ubiquitous characteristic of non linear systems with a high degrees of freedom. These systems exhibit universal statistical properties such as: chaotic behaviors, self-organized criticality, complexity, among others. There are many ways to study and interpret turbulence and turbulent phenomena. However, turbulent systems present some typical features. One of them is having large correlations between the variables of the system. This is the reason why in general turbulent systems are studied by simulations where, through first principle equations, it is possible to numerically analyze most of nonlinear or other effects. For example, as shown by Kolmogorov ^1^, a typical scaling exponent $-5/3$ of the energy fluctuations spectrum is usually observed in turbulent systems in the so called inertial range. Another typical property of turbulent systems is the non Gaussian Probability Density Function (PDF) of the fluctuations for some relevant quantities of the system such as velocity fluctuations or mean velocity increments, especially at small scales. A remarkable feature of these PDFs is the appearance of heavy tails^2–4^ which can be modeled by non-extensive distributions as $\kappa$-like distribution functions^5^.

Understanding how these properties emerge from the underlying governing equations is a fundamental challenge in different physics contexts, as aerodynamics^6,7^, hydrodynamics^8,9^, magnetohydrodynamics^10,11^, nematic fluids^12^, nonlinear optics^13^, quantum systems^14^, living matter^15^, medicine^16^ and more. Literature on these topics provides examples of different approaches for the study of turbulence. Namely, through experimental setups^3,17^, observational data^10,11^, particle^18^, fluid^19^, and Vlasov simulations^20^ of turbulent plasma systems, Langevin equations^21,22^, kinetic equations for weak turbulence^23^, kinetic equations for strong turbulence^24^, chaotic systems^25,26^, Shell Models^27,28^ and Coupled Map Lattices (CML)^25,29^.

CML are dynamical systems that model the behavior of non-linear systems and especially partial differential equations. They have been predominantly used to qualitatively study the chaotic dynamics of spatially extended systems. This includes the dynamics of spatio-temporal chaos where the number of effective degrees of freedom diverges as the size of the system increases. These are precisely the characteristics present in turbulent systems. Under this context, Beck ^25^ have implemented these ideas and proposed the CMLs as useful tool for the study of turbulence. In his study, the author proposes a CML model inspired in the Langevin equation, with a chaotic forcing, coupled with a $\beta$-model^30^ that describes the interaction between spatial scales. For an ensemble of eddies in different spatial scales, Beck ^25^ obtains the Velocity Distribution Function (VDF) of the radial component of velocity fluctuations. Among other characteristics, the results clearly show the appearance of non-thermal heavy tails in the VDF at different scales, or, in other words, when calculating the distribution function of velocity fluctuations it can be seen that it contain high-energy tails that decay as a power law, for values greater than one standard deviation around the mean, and thus moving away from the Maxwellian equilibrium.

As already mentioned, these non-thermal features can be modeled by non-extensive distributions. There are many works that relate turbulence with the representation of the system in terms of distributions functions different than the Maxwellian profile representing thermodynamic equilibrium^31,32^. Besides an ad-hoc fit, there is a strong relation between the shape of the VDF and the microscopic and macroscopic dynamics governing the system. In this sense, for systems out of equilibrium not only we could fit the PDF of turbulence quantities with non-extensive distributions (such as a $\kappa$-like distribution), but we can also understand the system from the point of view of non-equilibrium statistical mechanics^33,34^. In particular, we are interested in the relation between non-extensive statistical parameters and macroscopic parameters in a turbulent system. Under this context $\kappa$-distribution represent a robust and well-known framework to characterize and understand non-extensive statistical mechanics.

In this article we focus our attention on the formation of heavy tails in a turbulent flow. Considering a CML model similar to the proposed by Beck ^25^ with a white noise type forcing, we analyze the relation of a turbulent energy cascade on the spatial scale and the formation of $\kappa$-like distributions. We generate the steady state VDF of the elements of the model at each spatial scale, and show that the generated distributions can be well modeled by a $\kappa$-like distribution. Further, we observe a systematic relation between the $\kappa$ parameter, the spatial scale, and the Reynolds number of the system, and propose a closed relation on how they are related. For this, we have structured our study as follows: First we explain the basis of the CML model inspired by the Langevin force equation, to then expose the methodology for the treatment of the data obtained. Furthermore, we show how we modeled the relation between the spatial scale, the Reynolds number, and the $\kappa$ parameter of $\kappa$-like distribution, to finally summarize our findings and present the main conclusions.

## Model: coupled lattice Langevin based

Following the approach proposed by Beck ^35^, we consider a CML approach to model the system as a Langevin type form; i.e., the force equation for the elements of the system is the following:

1 $$\begin{aligned} \frac{dv}{dt} = - \gamma v + \zeta (t) \ , \end{aligned}$$

where $\gamma$ is the viscosity of the media, and $\zeta (t)$ is the noise forcing. In Eq. (1) *v* is given by

2 $$\begin{aligned} v(r,t) \equiv u_r( \mathbf{x}+ \mathbf{r},t) - u_r( \mathbf{x},t)\,, \end{aligned}$$

and denotes the difference of the radial component of the velocity field $\mathbf {u}$ at two points separated by a distance *r*. This parameter determines the observed spatial scale. To include the interaction between scales, the CML assumes that fully developed turbulence is characterized by the existence of eddies in different spatial scales. Each of this scales are labeled by an index *k*. In particular, the model contemplates eddies in the largest spatial scale in which turbulence is manifested, with size $\ell _0/2$, which are moving through a medium surrounded by other smaller eddies with size $\ell _0/2^{k}$ in the inertial range. We assume that the first scale with turbulent behavior ($k = 1$) is modeled by the Langevin force equation in (1), with a stochastic forcing given by

3 $$\begin{aligned} \zeta (t) = (\gamma \tau )^{1/2}\sum _{n = 1}^{\infty } x_n \delta \left( t - n\tau \right) \ . \end{aligned}$$

**Figure 1:**
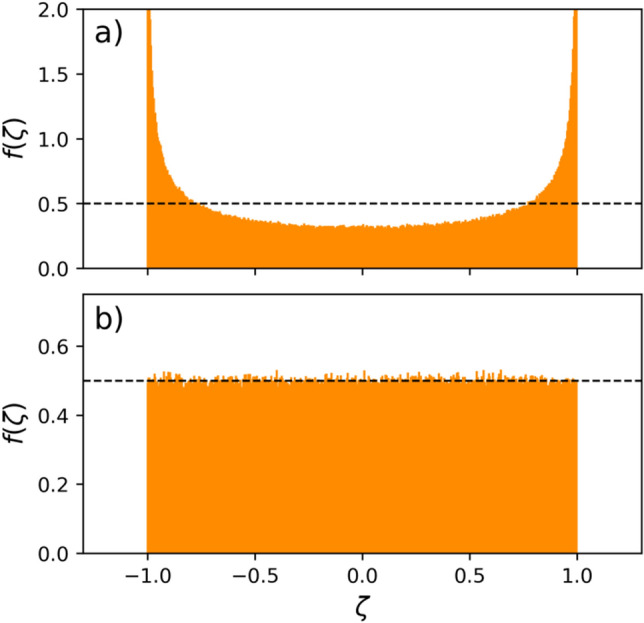
Comparison between the noise type probability density functions used in Ref. ^21^ (top), and the random distribution approach we follow here (bottom). Each panel shows the noise PDFs after $10^5$ time steps for two type of noises. We also remark a black dotted line as a reference for the 0.5 level.

Here $\tau$ is interpreted as the time average of “collisions” or interactions in the largest scale, which could be produced by an arbitrary driving force in the system, and $x_n$ is the normalized amplitude of fluctuations or noise. In the original derivation, in order to study the formation of heavy tails and asymmetric VDFs in turbulent flows, Beck ^25^ considers $x_n$ to follow the chaotic Ulam map, $x_{n+1} = 1 - 2x_n^2$, which keep the fluctuations in the range of − 1 to 1, with a probability distribution given by $f(\zeta ) = [\pi \sqrt{1 - \zeta ^2}]^{-1}$ (see Fig. 1a). As shown by Beck ^21^, the use of the chaotic Ulam map is necessary to model the chaotic forces that produces skewed VDFs in the flow. However, here we consider $x_n$ as a random variable with a uniform distribution between $-1$ to 1. As a comparison, Fig. 1 shows the the noise probability density function, $f(\zeta )$, used in Ref. ^21^ (top panel), and the random noise used in our model (bottom panel). We choose this noise over the normal (or Maxwellian) noise, to maintain the range of fluctuations as similar as in Ref. ^21^, but focusing only on the formation of heavy tails of the VDF.

Since the smallest timescale is $\tau$, it is convenient to discretize equation (1) using (3). Therefore, integrating the Langevin equation in a small time interval $\Delta t = \tau$, we obtain

4 $$\begin{aligned} v_{n+1} = \lambda v_{n} + \sqrt{\gamma \tau }\, x_n \ , \end{aligned}$$

where $v_n \equiv v(\tau n)$ and $\lambda = e^{-\gamma \tau }$. Further, to model the interactions between scales we consider the conservation of momentum between scales *k* and $k+1$. The basic assumption is that the momentum loss at level *k* serves as a driving force at level $k+1$. Moreover, daughter eddies at level $k+1$ gets only a random fraction $\xi _n^k$ of the momentum loss of the mother eddy at level *k*, and other part is dissipated. Thus, the momentum balance for the eddies at scale *k* is

5 $$\begin{aligned} m_k v_{n+1}^{(k)} = \lambda _k m_k v_{n}^{(k)} + \xi _n^{(k-1)} m_{k-1}\left( 1 - \lambda _{k-1} \right) v_{n}^{(k-1)} \ , \end{aligned}$$

where $m_k$ and $m_{k-1}$ are the masses of eddies at scales *k* and $k+1$, respectively, $\lambda _k = e^{-k\gamma \tau }$ is a constant damping at level *k*. In addition, following Ref. ^21^ we considered $\xi _n^{(k-1)}$ as a uniform random distribution with values between 0 and 1. This parameter can be understood as a random fraction of transferred momentum from the scale *k* to the next scale $k+1$. In other words, the term $m_{k-1}(1-\lambda _{k-1})v_n^{(k-1)}$ represent the lost of momentum by friction in the *k* scale that serves as a driving force for the next scale, $k+1$. As the friction at the scale *k* is produced by several smaller eddies, hence only a fraction of energy is transferred to one eddy in the next scale. It is important to mention that other choices for $\xi _n^{(k-1)}$ may be considered. However, as argued by Beck ^21^, those details do not significantly affect the properties of the VDFs in the stationary state, which is the focus of our study.

Then, combining Eqs. (4) and (5), in discrete form, we obtain the Coupled Lattice Langevin Based model, and its equations are given by

6 $$\begin{aligned} \begin{aligned} v_{n+1}^{(1)}&= \lambda _1 v_{n}^{(1)} + \sqrt{\gamma \tau }\, x_n \ , \\ v_{n+1}^{(k)}&= \lambda _kv_{n}^{(k)} + c_k\ \xi _n^{(k-1)} \left( 1 - \lambda _{k-1} \right) v_{n}^{(k-1)} \ . \end{aligned} \end{aligned}$$

Here the factor $c_k$ is the inverse of $\beta _k = m_k/m_{k-1}$, a characteristic parameter of the $\beta$-model ^30^, which represent the coupling between spatial scales, through the ratio between the masses of the eddies at the scales *k* and $k+1$, respectively. Here, such relation comes from equation (5). In addition, the value of $c_k$ is also related to the spread of the PDF at the scale *k*, which is related with the thermal speed of the system in the case of velocity fluctuations. As mentioned by Beck ^25^, the relation between the $c_k$ values and formation and properties of heavy tails in the PDF is weak. Therefore, for the sake of simplicity and to focus on the description of the tails of the PDFs, it is customary to select $c_k = c$, constant for all scales. Hence, here we consider $c_k = c=2$ for all scales, meaning that, on average, a mother eddy preferentially interacts with eddies what are half its mass. Moreover, $v^{(k)}_n$ can be interpreted as velocity of the center of mass of an eddy in the *k*-scale at time $n\tau$. Finally, The $\gamma \tau$ factor is the ratio between two characteristics time scales of the system. First we have $\tau$, the time scale of fluctuations in the large scale $\ell _0$, given by $\tau \sim \ell _0/w$, where *w* is the thermal velocity of the fluctuations. On the other hand, in the Langevin Equation $\gamma$ is the dynamic viscosity given by $\gamma \sim \nu _0/\ell _0^2$, with $\nu _0$ the kinematic viscosity. Then, we have $\gamma \tau \sim \nu _0/w\ell _0 \sim$ Re$^{-1}$. Namely, $\gamma \tau$ factor is inversely proportional to the Reynolds number of the fluid, Re ^21,25^. Therefore, as $\gamma \tau$ is the only free parameter of the model, in the CML, the dynamics of the system is determined by the level of turbulence of the media, measured by Re. The CML equations (6) represent a straightforward model to study the consequences of turbulence as a one dimensional system at different scales.

## Numerical analysis and results

To numerically solve the CML equations (6) we consider an ensemble of $N = 10^6$ eddies in each of the *k* scale, from $k = 1$ up to $k_\text {max} = 50$, and taking into account that the coupling constant between scales is $c_k = 2$. The election for this number allows us to solve the equations throughout several scales at different orders of magnitude, and was inspired by Beck’s results^25^, who considered $k_{max} = 17$. In addition, to test our model with different levels of turbulence (measured by the Reynolds number) we solve the equations using four different values for $\gamma \tau$, between $10^{-3.5}$ and $10^{-2}$.

As initial condition we select a delta function distribution for velocities centered at $v^{(k)}_0=0$, and evolve the CML equations in time until the system reaches a steady state with constant energy *E*. Namely,

7 $$\begin{aligned} \frac{dE}{dt} = 0, \quad \text {where} \quad E = \sum _{k = 1}^{k_\text {max}} \frac{1}{2}m_k \langle v^2 \rangle _k \ . \end{aligned}$$

**Figure 2:**
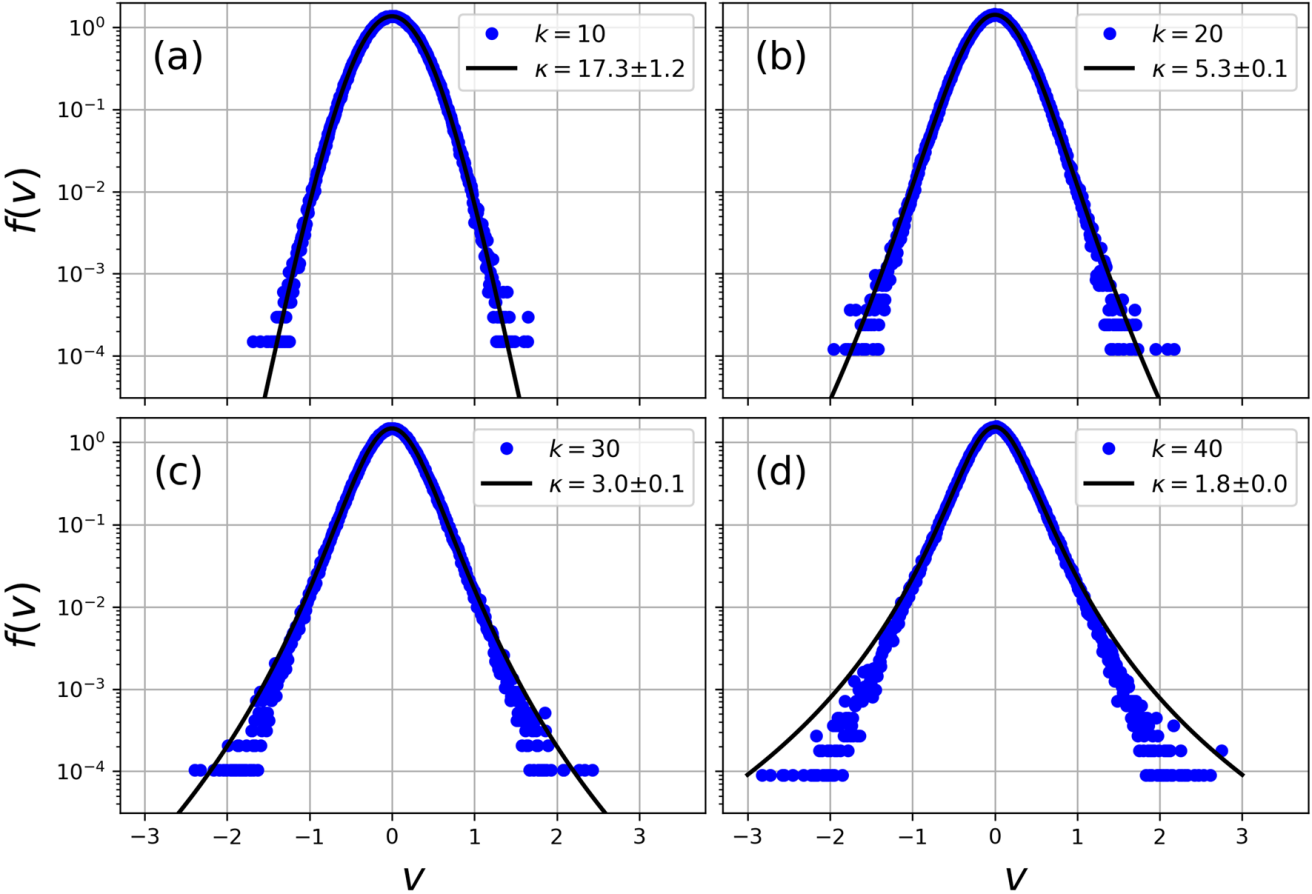
VDF of the Steady-state solution of the CML system in Eq. (6), at different *k*-scales and $\gamma \tau = 10^{-2.5}$. Blue dots represent the VDF of the data obtained with the simulations, and solid black lines the best fit using a $\kappa$-like distribution . In each panel the scale and the $\kappa$ parameter are: (a) $k = 10$, $\kappa = 17.3 \pm 1.2$, (b) $k = 20$, $\kappa = 5.3 \pm 0.1$, (c) $k = 30$, $\kappa = 3.0 \pm 0.1$, and (d) $k = 40$, $\kappa = 1.8 \pm 0.0$.

On the other hand, following observations of heavy tails^2–4^ and $\kappa$ distributions^5^ of velocity fluctuations in turbulent systems, at the steady state, we adjust the VDF in all *k* scales with a $\kappa$-like distribution ^36^

8 $$\begin{aligned} f_\kappa (v) = A_\kappa \left( 1 + \frac{1}{\kappa - 3/2}\,\frac{v^2}{w^2} \right) ^{-(\kappa +1)} \ , \end{aligned}$$

where $A_\kappa$, *w* y $\kappa$ are fit parameters. We had special attention to the relation between these quantities respect to the scale *k* for different values of $\gamma \tau$ between $10^{-3.5}$, and $10^{-1.5}$. $\kappa$-distributions correspond to a generalization of the Maxwellian distribution, widely used to model out-of-equilibrium systems in which the VDF exhibits large kurtosis due to powerlaw supra-thermal tails for higher energies (velocity). Under such context, Eq. (8), depending on the value of the $\kappa$ parameter the VDF will model the distribution with a quasi-thermal core (for $v\le \sqrt{k}w$), and power-law tails for larger velocities. The extent and energy of the tails increases with decreasing $\kappa$, and the VDF collapses to a Maxwellian in the limit $\kappa \rightarrow \infty$.

Figure 2 shows the VDF of steady-state solution of the CML system at $\gamma \tau = 10^{-2.5}$ and different *k*-scales at $k=$ 10, 20, 30 and 40. Blue dots represent the VDF obtained with the simulations, and solid black lines the best fit using Eq. (8). From the figure we note a good fit by (8) in the core of the VDF with a slight deviation in the tails of the distribution as we move in the *k*-scale, mainly due to counting statistics. In addition, it is clear that $\kappa$ decreases with increasing *k*, such that $\kappa =17.3\pm 1.2$ for $k=10$, and $\kappa =1.8$ for $k=40$.

**Figure 3:**
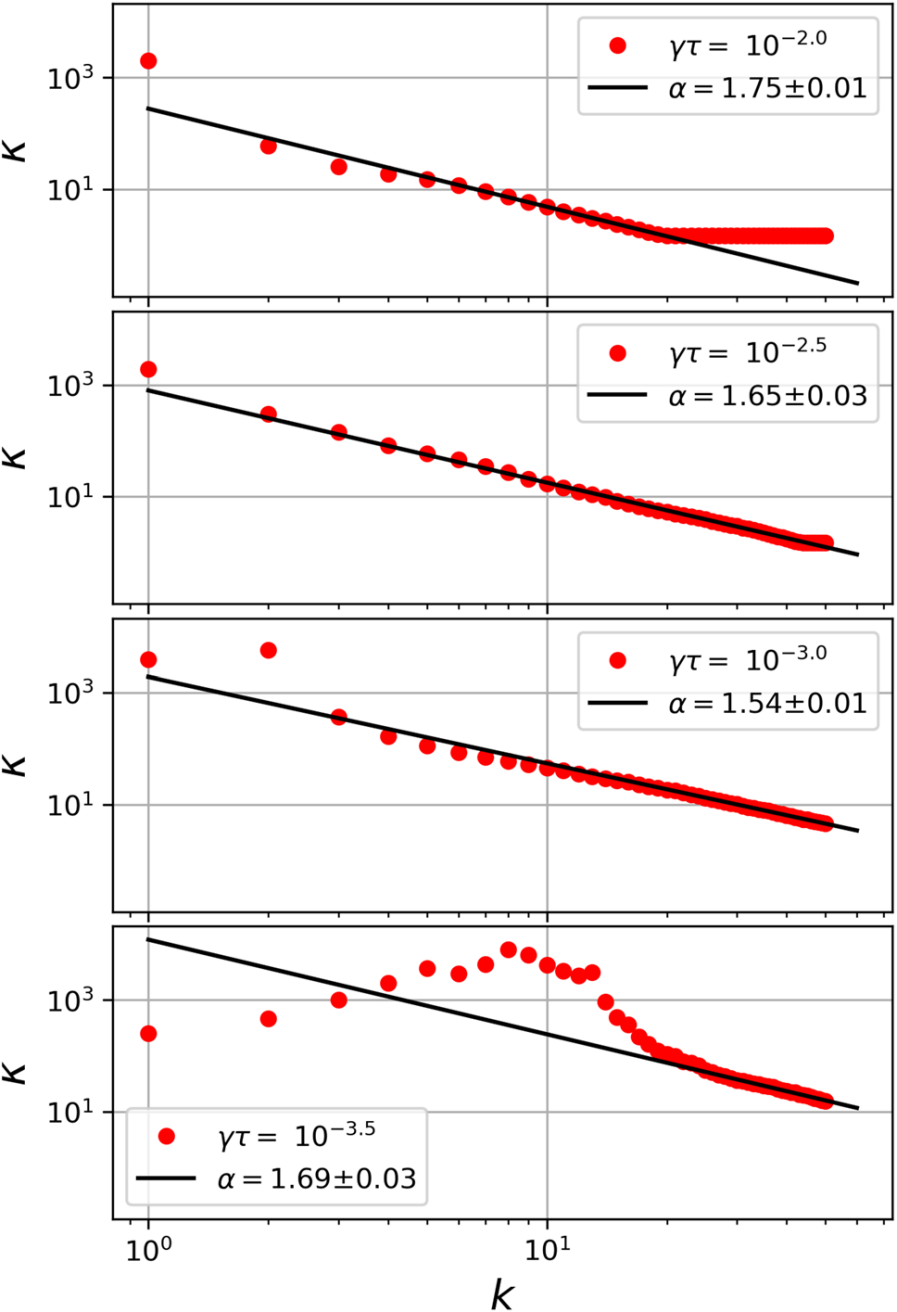
$\kappa$ parameter as a function of *k*-scale for different values of $\gamma \tau$ (10$^{-2.0}$, 10$^{-2.5}$, 10$^{-3.0}$, and 10$^{-3.5}$). For each case we made the respective fit using (9) and the points which best describe a power law.

To further characterize this behaviour, we performed a systematic analysis of the relation between $\kappa$, $\gamma \tau$ and the scale *k*. Figure 3 shows the adjusted $\kappa$ as a function of *k* for different values of $\gamma \tau$ (from top to bottom $\gamma \tau =$ 10$^{-2.0}$, 10$^{-2.5}$, 10$^{-3.0}$, and 10$^{-3.5}$, respectively). From the figure we can see that, in general, for all values of $\gamma \tau$, $\kappa$ exhibits large values for small *k*. This makes sense because we should expect a Brownian motion for the largest scale ($k=1$), represented by a Maxwellian VDF in the steady state, i.e. $\kappa \rightarrow \infty$. Therefore, it would be difficult to obtain an accurate value for $\kappa$. On the other hand, as we move on the *k*-scale, towards high values, $\kappa$ rapidly decreases with increasing *k*, up to the saturation scale (largest *k*) in which $\kappa \rightarrow 3/2$ (the smallest possible $\kappa$ value in our model). Therefore, as *k* increases, the $\kappa$ parameter is finite and smaller. In other words, as the scale size decreases the system moves away from a Maxwellian regime and the VDF is well represented by a $\kappa$-like distribution.

**Figure 4:**
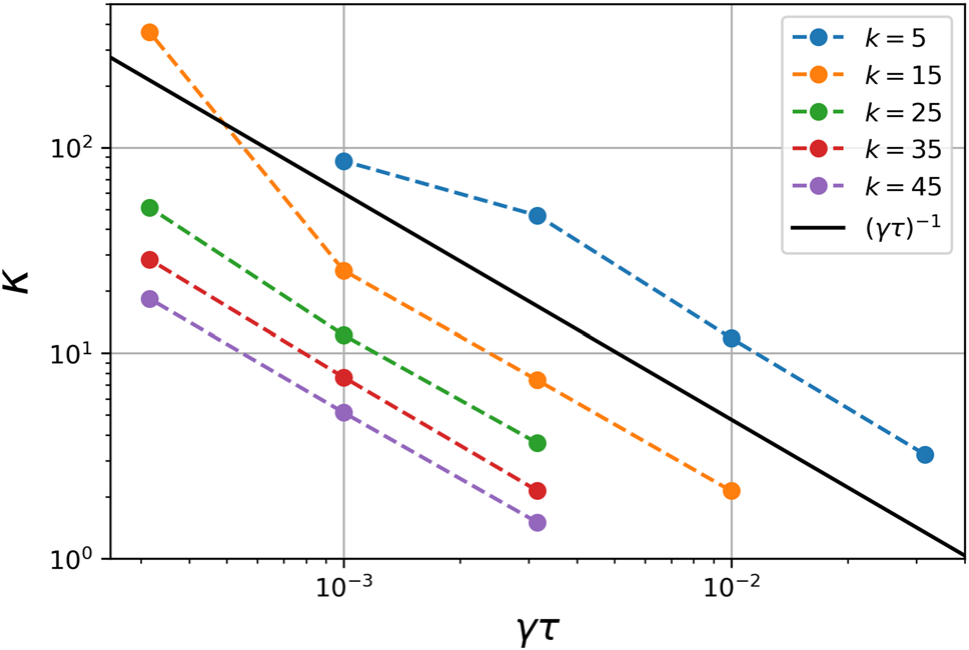
$\kappa$ parameter as a function of $\gamma \tau$ considering different values of *k* between $k=5$ and $k=45$. The solid black represents the curve $(\gamma \tau )^{-1}$, as a qualitative reference to show the quantitative correspondence among all the curves.

In addition, from Fig. 3 we observe a clear decaying power law behavior $\kappa \sim k^{-\alpha }$. For each value of $\gamma \tau$ the best power law is also included as a superposed black line in each panel. Remarkably, for all considered values of $\gamma \tau$, $\alpha \approx 5/3$. Furthermore, from the figure it is also possible to note how this power law behaviour also scales with the value of $\gamma \tau$, as, in general, $\kappa$ decreases with increasing $\gamma \tau$. However, from the Figure it is possible to recognize two regimes where the purposed power law model is no longer representative: first, for low values of *k* (or high spatial scales), given that the points are expected to be dispersed since the system tends to the Maxwellian state. As Maxwellian is the limit when $\kappa \rightarrow \infty$, when the system is more Maxwellian than Kappa distributed, the algorithm found high values for $\kappa$ (larger than $10^2$), and therefore there is low precision in the convergence of the fit. Second, for high values of *k* (small spatial scales), where the points saturate to a fixed value of 3/2. Since the $\kappa$-like fit we use has a singularity at this value, where the tails of the PDF seem too large to be described using a Kappa distributions. In such cases, other models may provide a better description of the PDFs. Finally, it is important to mention that the validity range in *k* also depends on the $\gamma \tau$ number, as shown in the panels of the aforementioned figure. We can see that as $\gamma \tau$ changes, the valid range clearly changes its position. The lower $\gamma \tau$ is, the higher and more shifted to the right (smaller scales) the range is. In other words, as the Reynolds number increases, smaller scales can be well represented by Kappa distributions.

On the other hand, the dependence of $\kappa$ with respect to the $\gamma \tau$ number can be seen in Fig. 4. The figure shows the value of $\kappa$ as a function of $\gamma \tau$ at different scales (different values of *k*), that also follows a power law behaviour. In this case, we have found that $\kappa \sim (\gamma \tau )^{-1}$ as shown by the superposed black line in the figure. In summary, we have found that the relation between $\kappa$, $\gamma \tau$ and *k* is given by $\kappa (k,\gamma \tau ) = \kappa _0 \ (\gamma \tau )^{-v}k^{-\alpha }$, where $\kappa _0$ is a constant parameter, $\nu \approx 1$, and $\alpha \approx 5/3$. Again, is worth mentioning that here we can also see dispersed points for $\kappa \sim 10^2$, which are out of the power law trend, a expected behaviour due to the low precision in the convergence of the fit when the PDFs approaches a Maxwellian shape.

Furthermore, considering the relation between $\gamma \tau$ and the Reynolds number, which previously we had mentioned is $\text {Re} \sim (\gamma \tau )^{-1}$, our numerical results indicate that

9 $$\begin{aligned} \kappa (k,\gamma \tau ) \sim \ \text {Re}\ k^{-5/3} \ , \end{aligned}$$

is the relation between $\kappa$, the Reynolds number and the space scale. Therefore, the equation (9) is a functional relation which is valid for a certain range of spatial scales, according to the Langevin based model purposed.

## Discussion and conclusions

In this paper we present a numerical study of the VDF generated in turbulent flows at different spatial scales, and different levels of turbulence. To do so, we used a Couple Lattice model, that considers turbulence as a coupled Langevin system at different scales. With such mode we found that the PDF of velocity fluctuations at different scales can be well represented by $\kappa$ distributions, and that the value of $\kappa$ depends on the scale *k* and the level of turbulence represented by $\gamma \tau$ (i.e. the Reynolds number).

Our numerical results show the relation between the spatial scale and the $\kappa$ parameter exhibits a power law behavior given by Eq. (9); i.e., when the Reynolds number grows, the $\kappa$ parameter also increases. This fact is expected since in a system with large enough Reynolds number, strong turbulence should inhibit the internal correlations leading to non Maxwellian PDFs. Remarkably, these results match with the observational study by Pollock et al. ^5^, where the Partial Variance of Increments (PVI) ^37^ of the velocity and magnetic field fluctuations (whose definitions are similar to (2)) were calculated in the context of space plasmas. In their study, the authors show that they obtained the respective PDFs of PVI, and showed that it generates distributions with heavy tails well fitted by $\kappa$-like distributions, with the value of $\kappa$ decreasing for decreasing spatial scale (larger *k*). It is important to note that the system studied in Refs. ^5,38^ is mainly a collisionless plasma, which also contemplates electromagnetic interactions. Even though the electromagnetic turbulence is beyond the scope of our study, the good agreement between our model and the results reported in Refs. ^5,38^ suggest a possible universal behaviour of the PVIs in turbulence, and also encourage us to extend our model to electromagnetic systems.

We have also found that the $\kappa$ parameter follows a scaling exponent $-5/3$. i.e., the value of $\kappa$ follows the same Kolmogov’s law, which might be not surprising for a turbulent flow. However, even though this is the same scaling behavior of the energy spectrum of the turbulence cascade in the inertial range, the fact that the shape of the high energy tails of the PDF for velocity fluctuations follows the same scaling is not a trivial result. This suggests that, indeed, turbulence may provide a connection between the kappa parameter to the spatial scale, and that $\kappa$ scales linearly with energy, which is also consistent with previous theoretical works. For example, Hasegawa ^38^ found a close expression for $\kappa$ as a function of energy, given by $\kappa = E_0/E_{T_e}$, where $E_0$ is a critical energy representative of the system, and $E_{T_e}$ is the thermal energy of the media. Thus, the CML approach may represent a way to find the implicit dependencies of the non-thermal (non Maxwellian) properties of a turbulent system with respect to others parameters, and their relation with the first principles statistical mechanics description of turbulent flows. In fact, as shown by Beck ^25^ by using other kind of noises in the stochastic term in equation (1), the CML has the potential to be extended or generalized to reproduce, not only the heavy tails, but also the skewness of the VDF that can also be found in turbulent flows.

That being said, the differences between the best fit using $\kappa$-distribution and large amplitude velocity fluctuations at high values of *k* (see Fig. 2) shows that other non-extensive distributions such as stretched exponential ^39–41^ or regularized $\kappa$-distributions ^42,43^ may be considered in order to extend the scope of the use of CMLs to characterize turbulent flows. Moreover, the CML also lacks nonlinear interactions between scales, or coupling between increments at different spatial locations either, all important effects to be considered to further characterize turbulence. In that sense, certain elements of shell models, as the non-local coupling, could be useful to improve the correspondence of the model with the characteristics of a turbulent system. For example, the GOY model^44–46^ provides multiscale statistics with many striking similarities with real turbulent flows.

However, even though we have considered a simplified CML model of a turbulent flow, our results show a non-trivial scaling relation between the shape of the VDF, and the level of turbulence of the media. From a numerical and Langevin theory point of view we have found a quantifiable relation between non-extensive parameter, such as $\kappa$, providing a relation between turbulence and the non-extensivity of the statistical mechanics of the system. In summary, on the basis of a simple numerical model of a turbulent case we have found that the PDFs of velocity fluctuations follow $\kappa$ distributions, and that there is a robust relation between the scaling of $\kappa$ and the Reynolds number of the flow. We expect these results to be useful to further characterize turbulent systems in different contexts, and our numerical predictions to be tested by observations or in experimental setups. We plan to address these aspects and other issues, expanding the scope of our model in subsequent works.

## Data Availability

The data that support the findings of this study are openly available in Zenodo at https://doi.org/10.5281/zenodo.4498354, reference number ^47^.
